# Tannic acid inhibits EGFR/STAT1/3 and enhances p38/STAT1 signalling axis in breast cancer cells

**DOI:** 10.1111/jcmm.13015

**Published:** 2016-11-15

**Authors:** Pramod Darvin, Youn Hee Joung, Dong Young Kang, Nipin Sp, Hyo Joo Byun, Tae Sook Hwang, Hema Sasidharakurup, Chi Ho Lee, Kwang Hyun Cho, Kyung Do Park, Hak Kyo Lee, Young Mok Yang

**Affiliations:** ^1^ Department of PathologySchool of MedicineInstitute of Biomedical Science and TechnologyKonkuk UniversitySeoulSouth Korea; ^2^ Amrita School of BiotechnologyAmrita Vishwa Vidyapeetham (Amrita University)KollamIndia; ^3^ Department of Food Science and Biotechnology of Animal ResourcesKonkuk UniversitySeoulSouth Korea; ^4^ National Institute of Animal Science, RDACheonanSouth Korea; ^5^ Department of Animal BiotechnologyChonbuk National UniversityJeonjuSouth Korea

**Keywords:** G1 arrest, p38/STAT1, STAT1 ser727, STAT3/BCl‐2 mitochondrial apoptosis, tannic acid

## Abstract

Tannic acid (TA), a naturally occurring polyphenol, is a potent anti‐oxidant with anti‐proliferative effects on multiple cancers. However, its ability to modulate gene‐specific expression of tumour suppressor genes and oncogenes has not been assessed. This work investigates the mechanism of TA to regulate canonical and non‐canonical STAT pathways to impose the gene‐specific induction of G1‐arrest and apoptosis. Regardless of the p53 status and membrane receptors, TA induced G1‐arrest and apoptosis in breast cancer cells. Tannic acid distinctly modulated both canonical and non‐canonical STAT pathways, each with a specific role in TA‐induced anti‐cancer effects. Tannic acid enhanced STAT1 ser727 phosphorylation *via* upstream serine kinase p38. This STAT1 ser727 phosphorylation enhanced the DNA‐binding activity of STAT1 and in turn enhanced expression of p21^Waf1/Cip1^. However, TA binds to EGF‐R and inhibits the tyrosine phosphorylation of both STAT1 and STAT3. This inhibition leads to the inhibition of STAT3/BCL‐2 DNA‐binding activity. As a result, the expression and mitochondrial localization of BCl‐2 are declined. This altered expression and localization of mitochondrial anti‐pore factors resulted in the release of cytochrome c and the activation of intrinsic apoptosis cascade involving caspases. Taken together, our results suggest that TA modulates EGF‐R/Jak2/STAT1/3 and P38/STAT1/p21^Waf1/Cip1^ pathways and induce G1‐arrest and intrinsic apoptosis in breast carcinomas.

## Introduction

Breast cancer (BCa) is a major cause of cancer‐related death with the highest prevalence than other cancers worldwide 1. Although insights and knowledge of cancer and its oncogenic mechanisms are abundant, treating or curing the disease is still a challenge. Natural chemicals are being tested for the safer management of human malignancies and to prevent the onset of cancers.

Polyphenols are plant‐derived secondary metabolites, recognized for their anti‐mutagenic, anti‐oxidant and anti‐cancer effects 2, 3. Tannins are water‐soluble polyphenols widely distributed in the plant kingdom, including food grains and fruits 4. Among the polyphenols, tannic acid (TA) is a hydrolysable tannin that yields glucose and gallic acid upon hydrolysis 5. Tannic acid has been used as a therapeutic agent for centuries 6. In recent years, research has attempted to elucidate the mechanistic aspects underlying the medicinal properties of TA, which has anti‐cancer properties by inducing apoptosis and controlling cancer cell proliferation 7. Tannic acid is well known for its radical scavenging and anti‐oxidant activities 8, 9.

Tannic acid has been reported to have high tyrosine kinase inhibition capacity. Strong inhibition on the tyrosine kinase activity of epidermal growth factor receptor (EGFR) and a weak inhibition on the P60 and insulin receptor tyrosine kinase were observed with TA treatment 10. Tyrosine kinase activity of TA is also observed with inhibition of CXCL12 (SDF‐1α)/CXCR4. In this case, TA served as an anti‐angiogenic factor 4. At very high concentration, TA showed inhibition on serine/threonine kinases including cAMP‐dependent protein kinases, protein kinase c and mitogen activated protein kinases 10. Tannic acid also has the ability to control various channels and pumps. The expression of PGP, MRP1 and MRP2 membrane efflux pumps were decreased upon TA treatment. Moreover, TA decreased calcein efflux 11.

Cancer is characterized by deregulated cell cycle progression. The cyclin‐dependent kinases (CDKs) are critical components of the cell cycle machinery, combining and activating D‐type cyclins. This CDK/cyclin D complex phosphorylates retinoblastoma (Rb) protein 12, leading to progression of the cell cycle. Cyclin‐dependent kinase/cyclin activity is controlled by CDK inhibitors 13, 14, which are divided into two families depending on the structure and target: INK4 and Cip/Kip 15, 16, 17. The p21^Waf1/Cip1^ and p27^Kip^ proteins mainly affect the D/E type cyclins 18 and prevent them from phosphorylating their targets. The major substrate for CDKs is Rb protein 19, 20. Phosphorylation of Rb leads to its inactivation, which activates E2F transcription factors, leading to the synthesis of protein machinery necessary for exiting the G1 phase into S phase 21, 22. Similarly, the activation of Sp1 through phosphorylation leads to the transcription of various other cell cycle components 23, 24. Evidence indicates that STAT1 and Sp1 synergistically act on target genes 25 and support one another in the transcription process.

Loss of control over the induction of apoptosis is another important aspect of cancer pathogenesis and is recognized as one of the major hallmarks of malignancies 26, 27. Apoptosis occurs mainly through two distinct mechanisms. One mechanism is as a result of the signals from internal factors, referred to as intrinsic apoptosis or the mitochondrial‐mediated pathway. Intrinsic apoptosis usually occurs with changes in Bcl‐2 and mitochondrial signals. The second mechanism is known as extrinsic apoptosis, or the death receptor pathway, in which specialized outer‐membrane death receptors are involved. In both mechanisms, effector caspases are activated and act as the key factors in the death event 28.

Evidence suggests that the Janus kinase 2/signal transducer and activator of transcription 3 (Jak2/STAT3) signalling pathway is associated with oncogenesis and the progression and metastasis of different cancers. Constitutively, active STAT3 is also observed in various malignant transformations, including that of the breast 29, head and neck 30, skin 31, ovaries 32, brain 33 and prostate 34. We have reported that STAT3 modulates VEGF through hypoxia inducible factor – 1α 35. STAT3 has direct transcriptional control over many genes, including survivin, VEGF, cyclin D1 and Bcl‐X_L_. Similarly, our studies with Methylsulfonylmethane have shown that inhibition of the Jak2/STAT3 pathway can inhibit breast tumour growth and pulmonary metastasis 36, 37, 38. Therefore, the inhibition of STAT3 should lead to the induction of apoptosis.

In this study, we analysed the role of TA in inhibiting proliferation and inducing G1 arrest and apoptosis in BCa cells. The molecular mechanism by which TA induces cell cycle arrest and apoptosis was studied with reference to STAT1/3. In support of our hypothesis, we found that both the enhancement of STAT1 ser727 phosphorylation and the inhibition of STAT1 tyr701 phosphorylation are key factors leading to G1 arrest upon TA treatment. Moreover, we found that TA modulates the EGF‐R/Jak2/STAT3 pathway, inducing mitochondrial apoptosis.

## Materials and methods

### Materials

Jak2, p‐Jak2 (Y1007/1008), p‐STAT1 (Y701), p‐STAT1 (S727), p‐STAT3 (Y705), p‐STAT3 (S727), Erk1/2, p‐Erk1/2 (Thr202/Tyr204), p38, p‐p38, p27^Kip^ and cyclin D1 antibodies were purchased from Cell Signaling Technologies (Beverly, MA, USA). STAT1, p21^Waf1/Cip1^, cyclin E, p53, CDK‐4, STAT3, Bax, Bcl‐2, Bcl‐X_L_, TATA binding protein (TBP), β‐actin antibodies and secondary antibody [rabbit, goat antimouse IgG‐horseradish peroxidase (HRP)] were obtained from Santa Cruz Biotechnology (Santa Cruz, CA, USA). The inhibitors of Jak2 (AG490), p38 (SB203580) and Erk1/2 (PD98059) were obtained from Sigma‐Aldrich (St. Louis, MO, USA). The inhibitor for TAK1 (5Z‐7‐oxozeaenol) was obtained from Millipore (Billerica, MA, USA). The inhibitors were dissolved in dimethyl sulphoxide (DMSO) for the experiments. Tannic acid (C_76_H_52_O_46_) for the analysis is purchased from Sigma‐Aldrich and dissolved in water for the treatment. The rhIL‐6 and rhIFN‐γ were purchased from Peprotech (Rocky Hill, NJ, USA) and rhIGF‐1 were obtained from Invitrogen (MD, USA). Tamoxifen (TAM) is purchased from Sigma Chemicals (St. Louis, MO, USA) and dissolved in DMSO. Gefitinib (GEF) was purchased from Cell Signaling Technologies.

### Cell culture and maintenance

The MCF‐7, T47D, SK‐BR 3 and MDA‐MB 231 cell lines were cultured and maintained in RPMI‐1640 or DMEM containing 10% Fetal bovine serum (FBS) and 1% penicillin/streptavidin (Gibco‐BRL, Grand Island, NY, USA). Unless otherwise specified, cells were grown in 10 cm dishes to ~80% confluence before being placed in serum‐free media for 18–24 hrs. Serum‐deprived cells were treated as specified in the figure legends.

### Cell proliferation studies using crystal violet assay

MCF‐7, T47D, SK‐BR 3 and MDA‐MB 231 cells were seeded in 6‐well plates and incubated overnight under ambient conditions. After 24 hrs incubation, the cells were treated with increasing concentrations of TA (20–100 μM) for 24 or 48 hrs. The cells proliferation was then analysed using crystal violet at 570 nm.

### Synergy quantification

The BCa cells were treated with different combinations of drugs for desired period. MCF‐7 cells were treated with ER‐α inhibitor TAM and TA individually and in combination. Similarly, MDA‐MB 231 cells were treated with EGFR inhibitor GEF and TA as individual agents and in combination. Combination of TAM and TA were done in the ratio of 1:10 and that of GEF to TA were 1:5. The combination index (CI) were calculated using the CompuSyn^™^ program (Biosoft, Ferguson, MO, USA) based on the Chou and Talalay principle 39. The program calculates the CI values and if the value is equal to 1 it is considered additive, a value higher to one is antagonist and lesser to one is considered synergistic. The data were represented as the fraction affected (Fa) with Fa = 1 representing 0% viability.

### Cell cycle analysis

The DNA content of BCa cells was determined using the BD Cycletest Plus DNA Reagent kit (BD Biosciences, San Diego, CA, USA) following the manufacturer's protocol. Briefly, approximately 5 × 10^5^ cells were permeabilized using trypsin buffer. The interaction of RNA with propidium iodide (PI) was neutralized by trypsin inhibitor and RNase buffer. The samples were then stained with PI and analysed using FACS Calibur (BD, Franklin Lakes, NJ, USA).

### Measurement of apoptosis

Annexin‐V‐FITC was used to quantify the percentage of cells undergoing apoptosis. The necrotic cells were counterstained with PI. The percentages of apoptotic cells were analysed by flow cytometry (BD FACScalibur, Franklin Lakes, NJ, USA). Cells treated with 10 μM Camptothecin served as a positive control.

### Western blotting

Jak2, p‐Jak2 (Y1007/1008), p‐STAT1 (Y701), p‐STAT1 (S727), p‐STAT3 (Y705), p‐STAT3 (S727), Erk1/2, p‐Erk1/2 (Thr202/Tyr204), p38, p‐p38, p27^Kip^ and cyclin D1 antibodies were purchased from Cell Signaling Technologies. STAT1, p21^Waf1/Cip1^, cyclin E, p53, CDK‐4, STAT3, Bax, Bcl‐2, Bcl‐X_L_, TBP, β‐actin antibodies and all secondary antibodies were obtained from Santa Cruz Biotechnology.

The BCa cell lines were treated with TA for pre‐determined times. For preparing nuclear extracts, Nuclear Extraction kit (Affymetrix, Santa Clara, CA, USA) and for mitochondria, Mitochondria Isolation kits (Thermo Scientific, Waltham, MA, USA) were used. For whole cell lysates, the cells were lysed on ice with RIPA lysis buffer containing 1× BD Baculogold protease inhibitor cocktail (BD Biosciences) and 1× PhosSTOP phosphatase inhibitors (Roche, Indianapolis, IN, USA). Equal amounts of protein were resolved by SDS‐PAGE and blotted onto a nitrocellulose membrane. The membranes were blocked with either 5% skim milk or bovine serum albumin (BSA) in TBS‐T buffer and then probed with primary antibody, followed by specific HRP‐conjugated secondary antibodies. The antibody was detected using the ECL plus detection kit.

### Semi‐quantitative RT‐PCR

All primers were from Bioneer (Daejeon, Korea). Total RNA was isolated from cells using the RNeasy Mini kit (Qiagen, Hilden, Germany) and reverse‐transcribed using the AccuPower RT‐premix kit (Bioneer) with oligo d(T) primers according to the manufacturer's instructions. PCR was performed with 2 μl of the reverse transcription product.

### Electrophoretic mobility shift assay

Breast cancer cells were grown to ~80% confluence and nuclear protein prepared using the Nuclear Extract Kit. STAT/DNA‐binding activity was detected by electrophoretic mobility shift assay (EMSA) using the EMSA Gel Shift kit (Panomics, Redwood City, CA, USA) according to the manufacturer's protocol. Briefly, the nuclear proteins were subjected to hybridization with a double‐stranded, biotin‐labelled oligonucleotide probe containing the consensus binding site for STAT1 (sense strand, 5′‐CATGTTATGCATATTCCTGTAAGTG‐3′) or STAT3 (sense strand, 5′‐CATGTTATGCATATTCCTGTAAGTG‐3′). The protein‐DNA complexes were resolved by 6% non‐denaturing PAGE, transferred to Pall Biodyne B nylon membrane (Pall Life Sciences, New Port Richey, FL, USA), and detected using streptavidin‐HRP and a chemiluminescent substrate.

### siRNA analysis

Approximately 1 × 10^5^ cells were cultured in a six‐well plate and transfected with ON‐TARGETplus SMARTpool siRNA targeting STAT1 or ON‐TARGETplus Non‐targeting siRNA (Dharmacon, Chicago, IL, USA) using Fugene‐6 (Roche, Basel, Switzerland) according to the manufacturers’ instructions. Following transfection, cells were treated with 60 μM TA for additional 24 hrs. Proteins were subsequently isolated and analysed by Western blotting.

### Transient transfection and generation of STAT1 S727A and STAT1 Y701F mutants

Approximately 1 × 10^5^ cells were cultured on six‐well plate and transfected with eGFP STAT1 S727A or eGFP STAT1 Y701F plasmids (gifts from Alan Perantoni 40, Addgene plasmid #12304 and #12302). GFP plasmid was used as a transfection control. The cells were transfected using X‐tremegene‐9 (Roche, USA) in Opti‐MEM (Gibco‐BRL) without antibiotics according to the manufacturer's instructions. Stable transfectants were selected using geneticin (G418, 800 μg/ml). The generated phospho‐mutants were treated in the presence or absence of 60 μM TA for 24 hrs and analysed for G1 phase arrest or protein expression.

### Chromatin immunoprecipitation assay

Chromatin immunoprecipitation (ChIP) assay was performed with the Imprint Chromatin Immunoprecipitation kit (Sigma‐Aldrich) according to the manufacturer's protocol. Briefly, MCF‐7 cells were fixed with 1% formaldehyde and quenched with 1.25 M glycine. The cells were suspended in nuclei preparation buffer, shearing buffer and sonicated under optimized conditions. The sheared DNA was centrifuged and the cleared supernatant used for protein/DNA immunoprecipitation. The clarified supernatant was diluted with dilution buffer (1:1) and 5 μl of the diluted sample removed as an internal control. The diluted supernatant was incubated in wells pre‐coated with STAT1 or STAT3 antibodies. Normal mouse IgG and anti‐RNA polymerase II were used as negative and positive controls respectively. The unbound DNA was removed by washing and the bound DNA collected by crosslink reversal using DNA release buffer containing proteinase K. The released DNA and the DNA from the internal control were purified using GenElute Binding Column G (Qiagen). The isolated chromatin was amplified using primers specific for the p21^Waf1/Cip1^ gene promoter region 41 or BCl‐2/STAT3 binding site. PCR amplification was also performed with optimized conditions: initial denaturation at 95°C for 5 min. and 40 cycles of 95°C for 30 sec., 58–60°C for 30 sec. and 72°C for 30 sec., followed by a final extension of 72°C for 10 min.

### Poly caspase assay

Caspase activation was studied using the Vybrant FAM poly caspase assay kit following the manufacturer's protocol. Briefly, BCa cells non‐induced and induced with TA or other chemical combinations were suspended at a concentration of 1 × 10^6^ cells/ml culture media. A 300 μl aliquot was mixed with 10 μl of 30× FLICA and incubated for 1 hr at 37°C and 5% CO_2_. The cells were then washed with 1× wash buffer and analysed using FACS Calibur.

### Statistical analysis

All experiments were repeated more than three times and the results expressed as mean ± S.E.M. Statistical analyses included the Student's *t*‐test or anova and were performed with SAS 9.3. A value of *P* < 0.05 was considered a significant difference.

## Results

### Tannic acid induces G1‐arrest and apoptosis in breast cancer cells and synergizes drug activities

**Figure 1:**
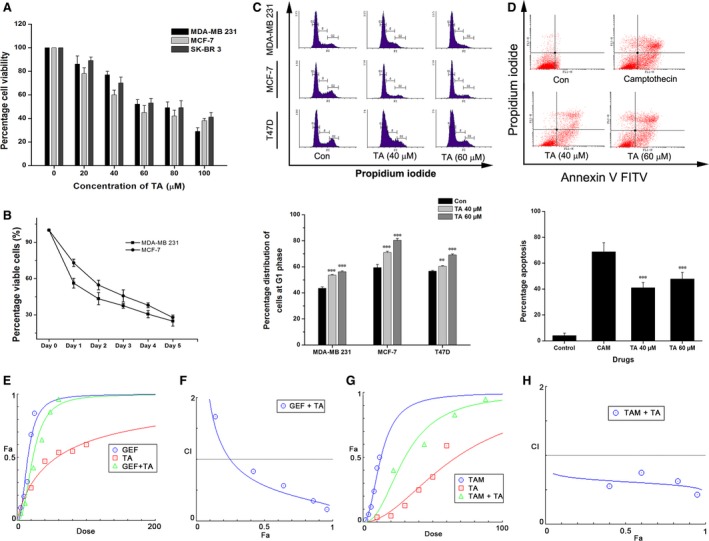
TA induces G1‐arrest and apoptosis in BCa cells. (**A**) TA induced proliferation inhibition on BCa cells. (**B**) The dose–response curve by growing the cells on 60 μM TA containing media for a maximum of 5 days. (**C**) The BCa cells were treated with increasing concentration of TA (40 and 60 μM). The G1‐arrest induced was measured using PI and (**D**) the apoptosis induced were measured using Annexin‐V‐FITC staining. (**E**) MDA‐MB 231 cells were treated with GEF and TA individually and in combination. The dose–response curve is plotted with Compusyn. (**F**) The combination index (CI) plot showing the synergy between GEF and TA. (**G**) MCF‐7 cells were treated with TAM and TA individually and in combination. The dose–response curve plotted with CompuSyn. (**H**) The CI‐plot showing the synergy between TAM and TA. Asterisks indicate a statistically significant increase by anova (**P < 0.01, ***P < 0.001).

Preliminary assays of proliferation inhibition revealed prominent growth inhibition of BCa cells with IC_50_ values of 50–70 μM/l upon treatment with TA for 24–48 hrs (Fig. 1A). Inhibition was more or less similar in basal type (MDA‐MB 231), luminal A (MCF‐7), and Her2‐expressing (SK‐BR 3) BCa subtypes. The dose–response curve showed very slow progression of proliferation inhibition, and for a treatment period of 5 days, it inhibited ~80% of BCa cell viability (Fig. 1B). The BCa cell lines MDA‐MB 231, MCF‐7, and T47D were exposed to TA at 40 or 60 μM for 24 hrs. Following TA exposure, the cells were stained using PI and the nuclear distribution analysed. Cells treated with 40 μM TA accumulated in the G1 phase with a decrease in the percentage of the cell population in the G2 phase (Fig. 1C). The percentage of cells in the G1 phase was higher for cells treated with 60 μM TA (56%, 79%, and 69%; *P* < 0.001) compared to 40 μM (54%, 71%, and 60%; *P* < 0.01, *P* < 0.001, and *P* < 0.001), showing concentration‐dependent G1 phase arrest in the BCa cells.

The dose–response curve showed a rapid inhibition of cell proliferation after 24 hrs. We have been suggested that this decrease was due to the induction of apoptosis. To verify this, triple‐negative MDA‐MB 231 BCa cells, which have mutated p53 and are highly metastatic, were treated with TA. In cells treated with TA, there was an accumulation of cells in the apoptotic phase (Fig. 1D). In cells treated with 60 μM TA, the apoptosis rate (45.47%, *P* < 0.001) was comparatively higher than that of the 40 μM‐treated cells (41.83%, *P* < 0.001), showing a concentration‐dependent induction of apoptosis. Altogether, the data confirm that TA induced G1 arrest, followed by apoptosis, in the BCa cells.

The combination effect of TA was studied in combination with EGFR inhibitor GEF and ER‐α inhibitor TAM. The TA in a combination ratio of 1:5 (GEF: TA) with GEF found synergistic. The CI values were calculated and it depicted a synergism (Fig. 1E and F). Similar to this, the treatment with TAM, at a ratio of 1:10 also induced synergic proliferation inhibition in ER‐α positive MCF‐7 BCa cells (Fig. 1G and H). The CI values were calculated using the CompuSyn^™^ software using the Chou and Talalay method (Table 1).

**Table 1:** Synergistic combination of TA with TAM and GEF

Cell line	Drug	Fa	Dm	m	*r*	CI value
MCF‐7	TA	0.5	66.6	1.95	0.94	0.619
	TAM		12.7	2.22	0.99	
	TAM+TA (1:10)		29.8 (2.7 + 27.1)	2.3	0.95	
MDA‐MB 231	TA	0.5	57.1	0.87	0.97	0.577
	GEF		15.37	2.37	0.93	
	GEF+TA (1:5)		22.7 (3.8 + 18.9)	2.44	0.97	

Synergism was measured with the Chou and Talalay method using the continuous approach with a fixed ratio. A CI value of <1.0 is considered as synergy. CI=1 is considered the additive effect and CI value >1 is called antagonistic activity. The parameters *Dm, m* and *r* are the slopes, antilog of *r* intercept, and the linear correlation coefficient of the median‐effect plot; which signifies the potency (IC_50_), the shape of the dose‐effect curve, and conformity of the data to the mass‐action law respectively. *Dm* and *m* values are used to calculate the CI value. TAM: Tamoxifen; TA: Tannic acid; GEF: Gefitinib; Fa: Fractional inhibition; CI: Combination Index.

### Tannic acid binds and inhibits EGF‐R activity

**Figure 2:**
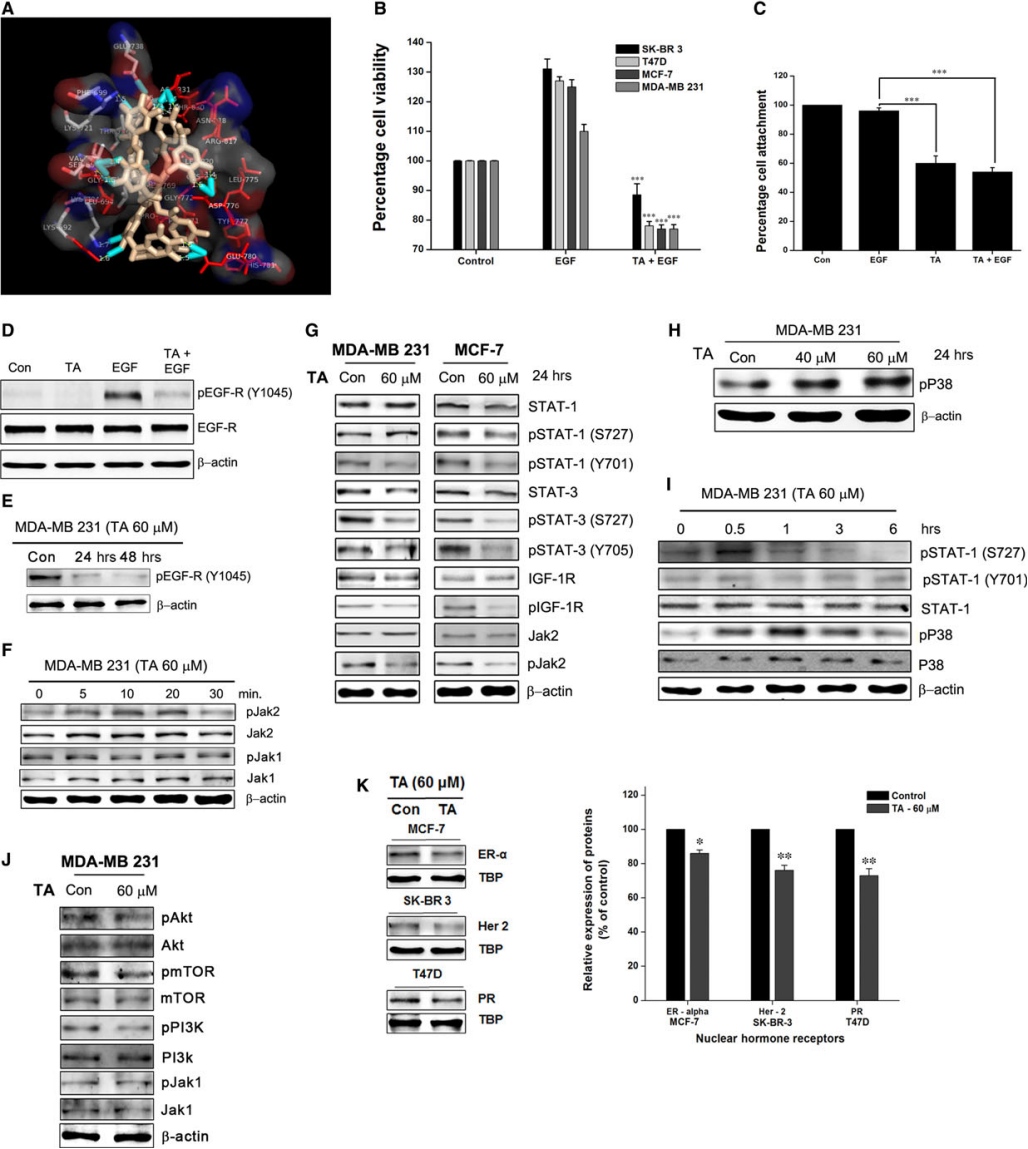
TA binds and inhibits EGF‐R signalling. (**A**) Binding of TA to the ATP‐binding domain of EGF‐R determined through molecular docking using Autodock vina. (**B**) BCa cells pre‐treated or not with 60 μM of TA for 4 hrs and treated with or without 10 ng/ml of EGF. The proliferation assessed using crystal violet staining method. (**C**) The BCa cells pre‐treated or not with TA (60 μM) were seeded in collagen coated plates with or without EGF (10 ng/ml). Following incubation, the cell attachment with the matrix in the presence of EGF and TA were analysed. (**D**) BCa cells pre‐treated with or without TA were induced with EGF. The EGF‐R phosphorylation was analysed using western blotting, and (**E**) The inhibition lasts for 48 hours after the withdrawal of TA. (**F**) Inhibition of EGF‐R auto‐phosphorylation inhibited the Jak2 phosphorylation, and (**G**) the STAT1 and STAT3 tyrosine phosphorylation. (**H**) TA enhanced the phosphorylation of serine kinase p38 in a concentration‐dependent manner. (**I**) Time dependent analysis showed an early time serine phosphorylation of STAT1 and p38. (**J**) TA inhibited the phosphorylation of PI3K‐Akt and mTOR in MDA‐MB 231 cells. (**K**) TA significantly inhibited multiple nuclear hormone receptors in BCa cells. Asterisks indicate a significant increase or decrease by *t*‐test (*P < 0.05, **P < 0.01, and ***P < 0.001).

To decode the molecular mechanism underlying the effects of TA on BCa cell proliferation, we tested the binding ability of TA with EGF‐R, Jak1, Jak2, IR and IGF‐1R. Molecular docking was performed with an autodockvina platform. Tannic acid docked with the ATP binding site of EGF‐R, Jak1, Jak2, IR and IGF‐1R. The study showed direct binding of TA with the receptor (Fig. 2A). The proteins were sorted based on their binding affinity and we saw that EGFR has the highest binding affinity with TA and IGF‐1R has the least binding affinity. Next to EGFR, Jak2 has the second highest binding affinity following IR and Jak1 respectively (Fig. S1). We tested the role of TA/EGF‐R binding in the EGF signalling axis and cell proliferation. Pre‐inhibition of EGF‐R with TA inhibited EGF‐induced cell proliferation (Fig. 2B) and cell‐matrix attachment (Fig. 2C, *P* < 0.001) in BCa cells. Molecular docking studies showed that various amino acids responsible for receptor auto‐phosphorylation were involved in TA/EGF‐R binding, suggesting that the TA binding will eliminate the kinase function of the receptor. Western blotting studies revealed that TA inhibited the auto‐phosphorylation of EGF‐R (Fig. 2D) and the EGF‐R associated tyrosine kinase Jak2 (Fig. 2F and I). Tannic acid mediated inhibition EGFR lasted for a period of 48 hrs (Fig. 2E). Moreover, the role of PI3K‐Akt‐mTOR phosphorylation and activation are dependent on the EGF singling and play major role in the malignant transformation through the induction of drug resistance, metastasis and inhibition of apoptosis 42. So we analysed the inhibition of PI3K‐Akt‐mTOR pathway as the TA has high degree of inhibition on EGFR. Tannic acid inhibited the PI3K‐Akt‐mTOR phosphorylation in MDA‐MB 231 cells (Fig. 2J).

### Tannic acid modulates canonical and non‐canonical STAT activation

STAT signalling molecules are phosphorylated and activated either by canonical or by the non‐canonical pathway. Tannic acid‐treated BCa cells revealed inhibition of Jak2 phosphorylation (Fig. 2F and G). Concurrent with the inhibition of Jak2, we observed that both STAT1 and STAT3 tyrosine phosphorylation were also inhibited. Interestingly, the total STAT1 and STAT1 ser727 phosphorylation were enhanced. In addition, the phosphorylation of IGF‐1R was inhibited, pointing to its ability to regulate the IGF‐1 signalling pathways (Fig. 2G). The concentration of phosphorylated p38 increased with TA treatment in a concentration‐dependent manner, suggesting the role of p38 serine kinases in STAT1 ser727 phosphorylation (Fig. 2H). An increase in serine phosphorylation was observed 30 min. after TA treatment and sustained up to 1 hr (Fig. 2I). The transient increase in STAT1 serine phosphorylation was consistent with the STAT1 expression levels in untreated samples. STAT1 tyrosine phosphorylation was inhibited at 30 min. and consistent up to at least 24 hrs (Fig. 2G). The level of non‐phosphorylated STAT1 remained unaltered until 6 hrs (Fig. 2I) and increased after 24 hrs (Fig. 2G).

**Figure 3:**
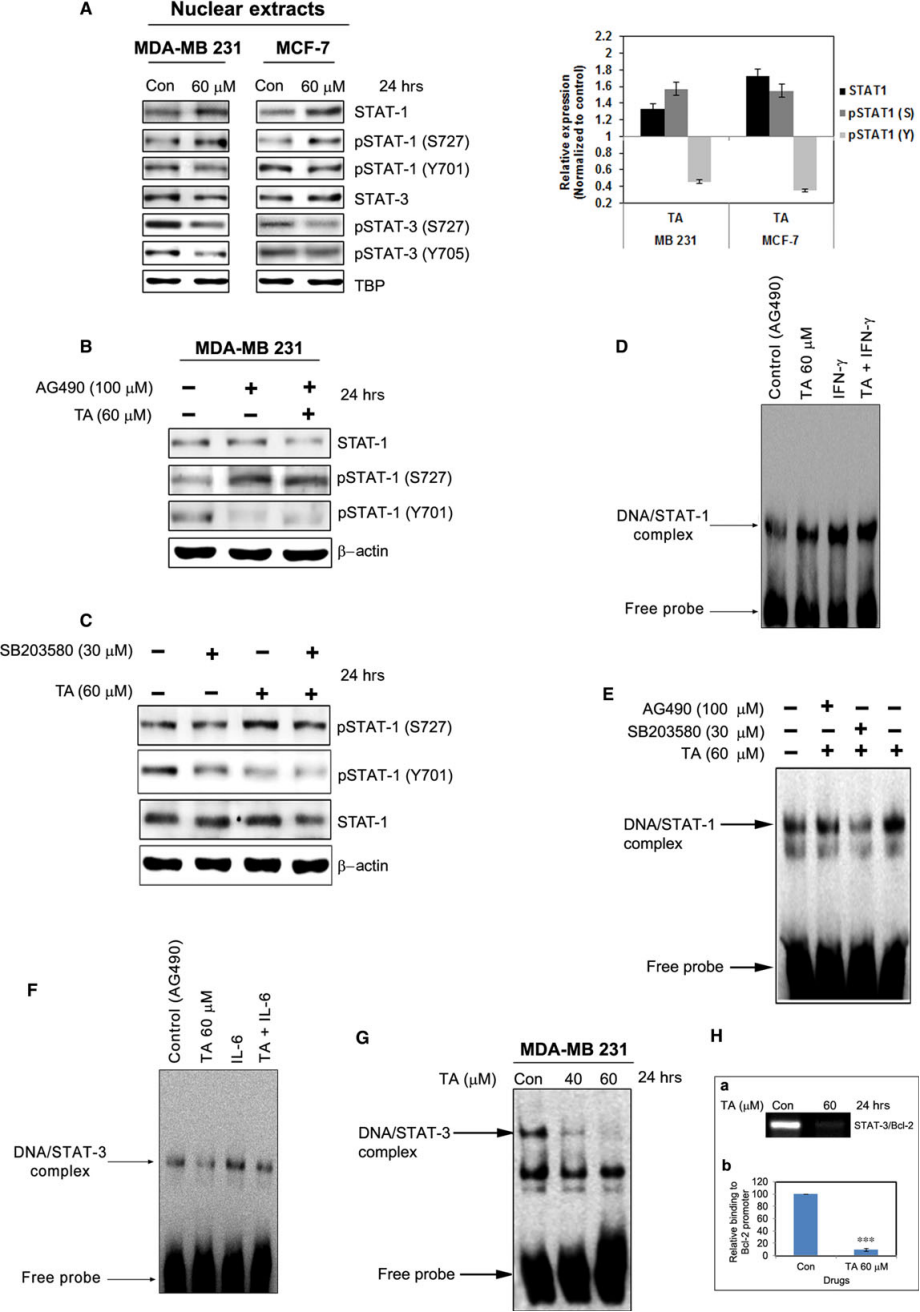
TA inhibits STAT1/3 phosphorylation and nuclear translocation. (**A**) BCa cells were treated with or without TA for 24 hrs and the nuclear extracts were prepared and analysed for the translocation of total as well as phosphorylated STAT1 and STAT3. (**B**) The BCa cells were treated with the Jak2 inhibitor; AG490 or (**C**) p38 inhibitor; SB203580prior to TA treatment and the phosphorylation of STAT1 were studied. (**D**) The BCa cells were pre‐treated with either AG490 or SB203580 prior to TA treatment and the STAT1/DNA binding activities were studied using EMSA. (**E**) BCa cells were treated with TA for 24 hrs and the STAT3/DNA binding activities were studied using EMSA or (**F**) the chromatin were prepared and analysed for the ability of STAT3 to bind BCl‐2 gene promoter using ChIP assay. (**G**) BCa cells were treated with AG490, TA, IL‐6 (Positive control for STAT3 signalling) and TA together with IL‐6 and the nuclear extracts were prepared and the STAT3/DNA binding were studied. (**H**) BCa cells were treated with AG490, TA, IFN‐γ (Positive control for STAT1 signalling) and TA together with IFN‐γ and the nuclear extracts were prepared and the STAT1/DNA binding were studied. Asterisks indicate a significant decrease by *t*‐test (***P < 0.001).

The nuclear extracts exhibited reduced STAT3 phosphorylation and STAT1 tyrosine phosphorylation, whereas the levels of total STAT1 and phosphorylated STAT1 ser727 were enhanced (Fig. 3A). Inhibition of Jak2 did not affect the TA‐induced STAT1 serine phosphorylation, though it inhibited tyrosine phosphorylation (Fig. 3B). Inhibition of p38 using SB203580 inhibited the TA‐induced STAT1 serine phosphorylation, confirming it as the upstream serine kinase (Fig. 3C). This inhibition did not affect tyrosine phosphorylation or total STAT1 levels. Gel shift analysis revealed TA increased the DNA‐binding activity of STAT1 (Fig. 3D). To resolve the role of Jak2 and p38 in STAT1/DNA‐binding activities, Jak2 and p38 were inhibited prior to TA treatment and gel shift analysis performed. The STAT1/DNA‐binding activity was partially inhibited with the inhibition of p38, but Jak2 inhibition resulted in no change in STAT1/DNA‐binding activity (Fig. 3E).

In contrast to the enhancement in STAT1/DNA binding, the DNA‐binding activities of STAT3 were inhibited (Fig. 3F). This inhibition was concentration‐dependent; at a concentration of 60 μM TA, DNA binding was almost completely eliminated (Fig. 3G). We performed ChIP assays to determine the effect of TA treatment on the STAT3/BCl‐2 gene promoter binding activity. As shown in Fig. 3H, the relative binding of STAT3 to the promoter was significantly inhibited after 36 hrs of TA treatment (*P* < 0.001).

### Canonical and non‐canonical pathways mediate distinct functions

**Figure 4:**
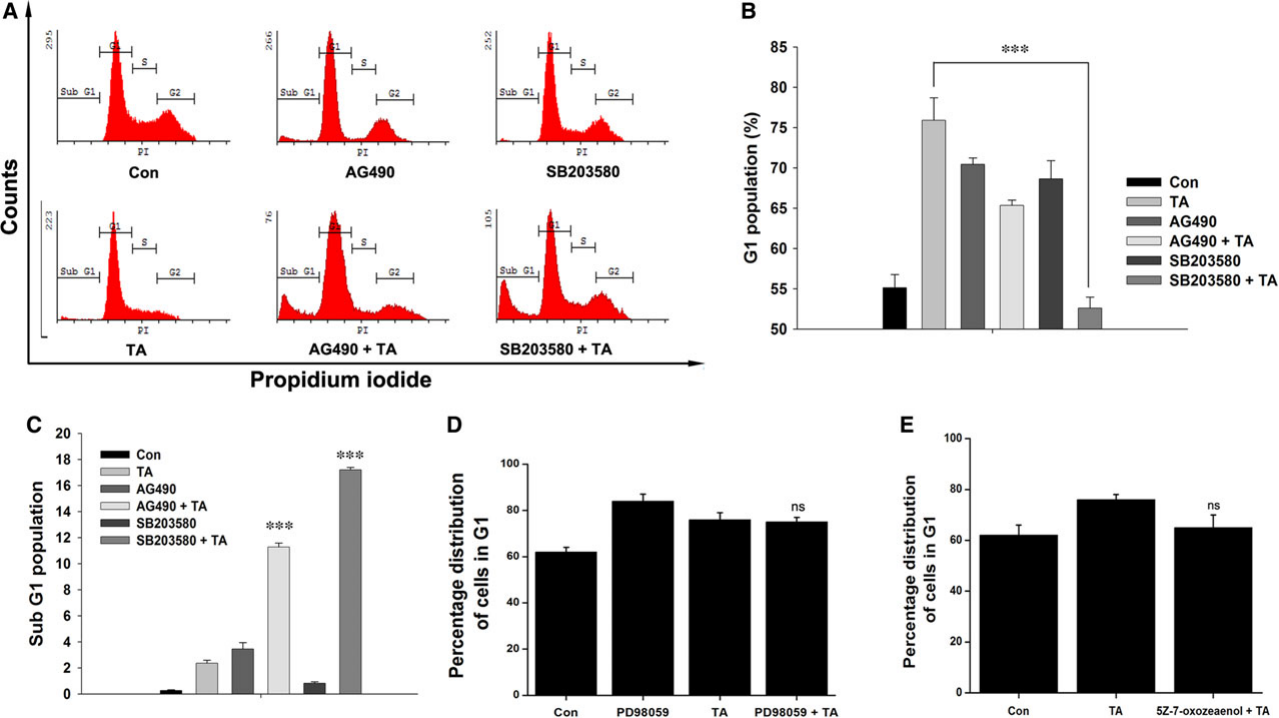
Tannic acid induces G1 arrest through p38/STAT1. (**A**) MCF‐7 cells were pre‐treated with AG490 or SB203580 for 1 hr followed with 60 μM TA for 24 hrs and analysed cell cycle distribution. (**B**) Percentage of cells accumulated on G1‐phase and (**C**) Sub‐G1 (apoptosis) phase up on TA treatment. (**D**) Erk1/2 were inhibited by pre‐treatment with PD98059 for 1 hr followed with 60 μM TA for 24 hrs and analysed cell cycle distribution. (**E**) TAK1 were inhibited by pre‐treatment with 5Z‐7‐oxozeanol for 1 hr followed with 60 μM TA for 24 hrs and analysed cell cycle distribution. The data shown are representative of three independent experiments with triple analysis. Asterisks indicate a significant increase or decrease by *t*‐test (ns ‐ Non significant and ***P < 0.001).

To confirm that TA acts by inhibiting the canonical EGF‐R/Jak2/STAT pathway and stimulating the non‐canonical p38/STAT1 pathway, cells were pre‐treated with SB203580 for 1 hr and exposed to TA. STAT1 ser727 phosphorylation (Fig. 3C) and TA‐induced G1 arrest were reduced (Fig. 4A and B). Furthermore, p38 inhibition increased the sub‐G1 population in the presence of TA (Fig. 4A and C). Western blot of TA‐treated BCa cells pre‐treated with or without AG490 revealed that the induced phosphorylation of STAT1 ser727 occurred independently of Jak2 (Fig. 3B), but STAT1 tyr701 phosphorylation was dependent on Jak2. Specific inhibition of Jak2 using AG490 did not influence TA‐induced G1 arrest (Fig. 4A and B), but it increased the sub‐G1 population, suggesting increased apoptosis (Fig. 4A and C). Thus, non‐canonical STAT1 ser727 phosphorylation plays a significant role in the induction of G1 arrest, and TA induces apoptosis in the absence of STAT1 ser727 phosphorylation. In contrast, the inhibition of canonical Jak2/STAT3 tyrosine phosphorylation leads to the induction of apoptosis. The involvement of multiple serine kinases was checked for their role in p38/STAT1 mediated G1 arrest. The specific inhibition of Erk1/2 (Fig. 4D) and TAK1 (Fig. 4E) showed no significant role in the TA mediated G1 arrest.

### STAT1 induces G1 arrest

**Figure 5:**
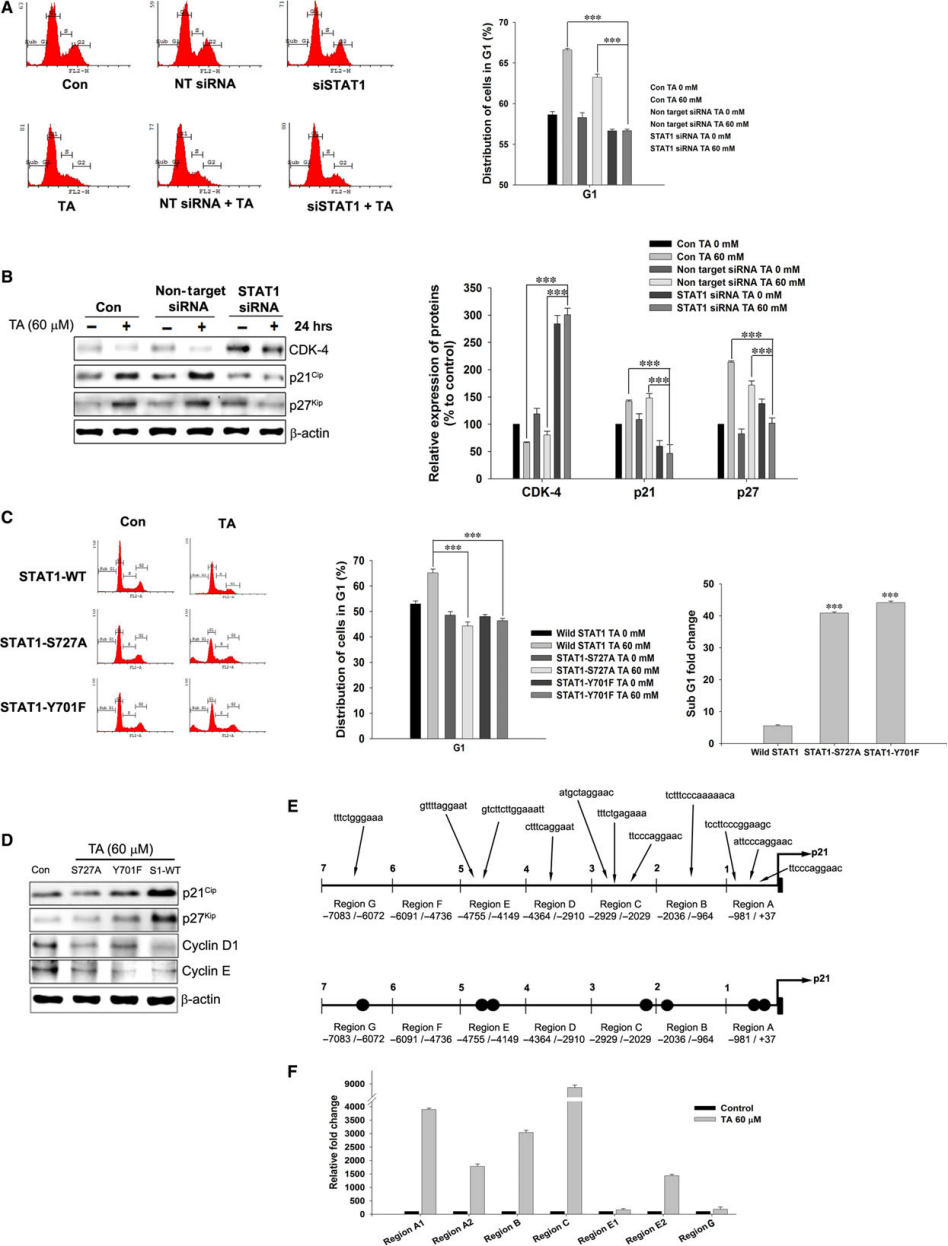
STAT1 is a critical mediator of TA induced G1‐arrest. (**A**) Specific knock‐down of STAT1 gene using ON‐TARGETplus SMART pool STAT1 siRNA. The cells were treated with or without using TA 60 μM and analysed the G1‐arrest. (**B**) STAT1 knock‐down followed with or without treatment of TA 60 μM. The samples were analysed for p21^Waf1/Cip1^, p27^kip^, and CDK‐4 expressions. (**C**) STAT1 phospho‐mutants were prepared and treated with TA for 24 hrs. The distribution of nucleus in G1 and sub‐G1 phase was analysed by PI staining. (**D**) The expression of p21^Waf1/Cip1^, p27^kip^, Cyclin D1 and Cyclin E in STAT1 phospho‐mutants treated with TA. (**E**) *In silico* screening for STAT1 binding domains on human p21^Waf1/Cip1^promoter. (**F**) STAT1 binding affinity to multiple binding regions on p21^Waf1/Cip1^ gene promoter. Fold changes were calculated using the binding affinity of positive control RNA pol II. Asterisks indicate a significant increase or decrease by *t*‐test (***P < 0.001).

Knocking down STAT1 led to an escape from TA‐induced G1 arrest and almost eliminated the capacity of TA to induce G1 arrest (Fig. 5A, *P* < 0.001), confirming STAT1 as a critical mediator of TA‐induced G1 arrest. Moreover, the STAT1‐silenced cells had elevated CDK4 and decreased p21^Waf1/Cip1^ and p27^Kip^ expression. The inhibition of G1 phase modulators was STAT1–dependent, with significant recovery upon STAT1 silencing (Fig. 5B, *P* < 0.001). To determine whether STAT1 ser727/tyr701 phosphorylation plays a significant role in the induction of G1 arrest, we made STAT1 phosphorylation mutants (STAT1 ser727ala and STAT1 tyr701phe). These phospho‐mutants exhibited recovery from TA‐induced G1 arrest with significant induction of apoptosis (Fig. 5C, *P* < 0.001). In addition, TA‐induced enhancement of p21^Waf1/Cip1^ and p27^Kip^ expression was lost in the phospho‐mutants (Fig. 5D). Collectively, the data confirmed the role of STAT1 as a critical mediator of TA‐induced G1 arrest, and a loss of STAT1 function in the presence of TA induces apoptosis.

### STAT1 binds to the human p21^Waf1/Cip1^ gene promoter

Using ChIP assay, we investigated whether STAT1 directly binds the p21^Waf1/Cip1^ promoter. Based on previous reports 41, primers were synthesized to assess STAT1 binding to the human p21^Waf1/Cip1^ gene locus up to −7 kb upstream of the transcription start site. *In silico* screening revealed 11 putative STAT1 binding sites in the p21^Waf1/Cip1^ gene promoter (Fig. 5E). Screening the entire −7 kb region revealed seven putative STAT1 binding sites out of 11 binding sites investigated (Fig. 5E). Region A (−981 to +37) had two binding sites with 1.7‐ and 4.5‐fold increases in STAT1 binding activity respectively. Region C (−2929 to −2029) also had a 4.2‐fold increase in chromatin upon treatment with TA compared to the positive control (Fig. 5F).

### Tannic acid inversely modulates cell cycle regulators

**Figure 6:**
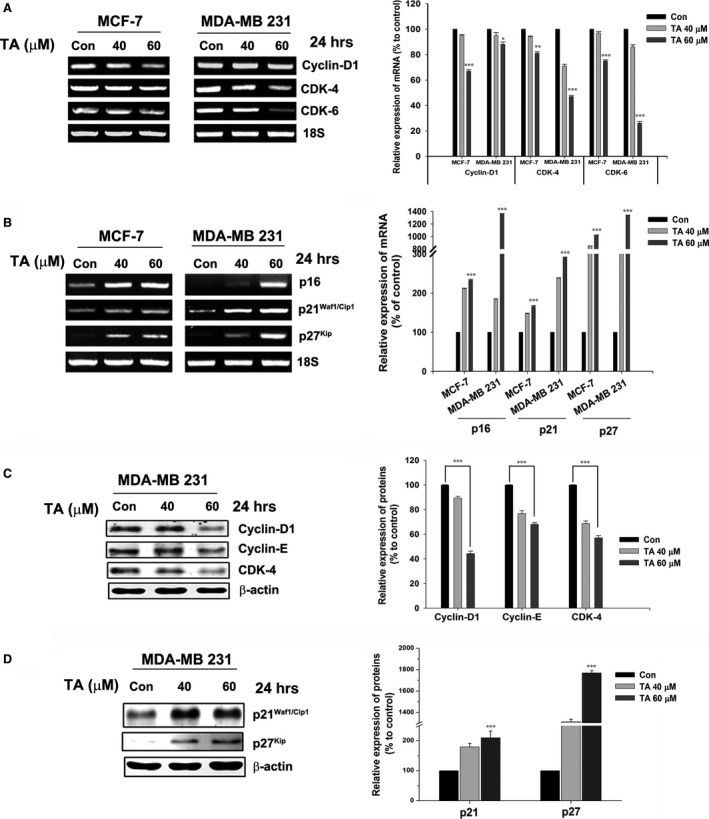
TA modulates the expression of cell cycle regulators. BCa cells were treated with increasing concentration of TA for 24 hrs. (**A**) Total RNA isolated and transcriptional regulation of positive modulators; Cyclin D1, CDK‐4, CDK‐6 and (**B**) negative modulators; p16^Ink4a^, p21^Waf1/Cip1^ and p27^Kip^ of the cell cycle were analysed. (**C**) The whole cell lysate is prepared and translational regulation of positive modulators; Cyclin D1, Cyclin E, CDK‐4 and (**D**) negative modulators; p21^Waf1/Cip1^ and p27^Kip^ of the cell cycle were analysed using western blotting. Asterisks indicate a significant increase or decrease by *t*‐test (*P < 0.05, **P < 0.01, and ***P < 0.001).

Tannic acid treatment dose‐dependently inhibited the expression of positive modulators of the cell cycle, cyclin D1, cyclin E, CDK4 and CDK6 both transcriptionally (Fig. 6A) and translationally (Fig. 6C). Transcriptional regulation of CDK4 and CDK6 followed a similar in MCF‐7 and MDA‐MB 231 cells (Fig. 6A), regardless of their p53 status. MCF‐7 cells exhibited prominent transcriptional inhibition of cyclin D1, whereas less significant inhibition was observed in MDA‐MB 231 cells (Fig. 6B). However, cyclin D1, cyclin E, and CDK4 exhibited significant inhibition at the translational level (Fig. 6C, *P* < 0.001).

Treatment with TA intensified the expression of negative regulators of cell cycle, p21^Waf1/Cip1^ and p27^Kip^ both transcriptionally (Fig. 6B) and translationally (Fig. 6D). Although the fold changes (2‐ and 17.6‐fold increase in p21^Waf1/Cip1^ and p27^Kip^) were different between these two cell lines (Fig. 6D), a similar pattern of p21^Waf1/Cip1^ enhancement was observed.

### Tannic acid regulates the expression and localization of apoptotic factors

**Figure 7:**
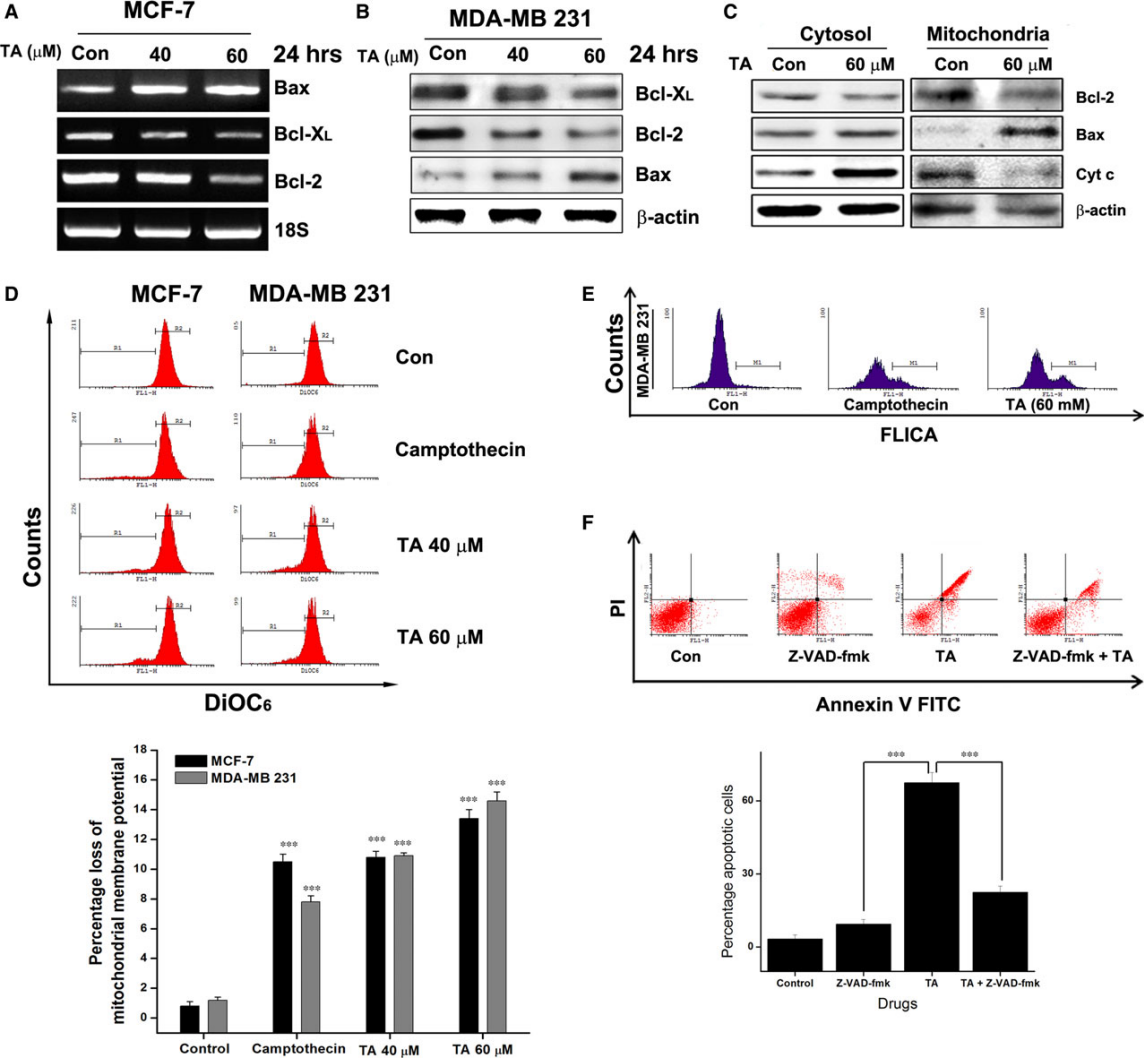
TA induces intrinsic apoptosis in BCa cells. (**A**) MCF‐7 cells were treated with TA for 24 hrs, the total RNA isolated and subjected to RT‐PCR analysis. (**B**) Western blotting of TA treated MDA‐MB 231 cells showing the inhibition of BCl‐X_L_, BCl‐2, and Bax. (**C**) Mitochondrial and cytosolic compartmentalization analysis of BCl‐2, Bax and Cytochrome c (**D**) The loss of mitochondrial membrane potential evaluated using DiOC_6_ staining. Asterisks indicate a significant increase or decrease by anova (***P < 0.001). (**E**) MDA‐MB 231 cells were treated with TA and stained with FAM‐FLICA poly caspase stain. (**F**) MDA‐MB 231 cells pre‐treated or not with caspase inhibitor (Z‐VAD‐fmk) were treated with TA. The cells undergoing apoptosis were detected. Asterisks indicate a statistically significant decrease by *t*‐test (***P < 0.001).

The inhibition of the binding of STAT3/Bcl‐2 gene promoter indicates the inhibition of its own expression. RT‐PCR studies were carried out in MDA‐MB 231 cells treated with increasing concentrations of TA. As expected, TA exposure led to the down‐regulation of Bcl‐2 and Bcl‐X_L_, both transcriptional (Fig. 7A) and translational level (Fig. 7B). However, the expression of Bax increased with TA concentration (Fig. 7A and B). Mitochondria isolated from the TA‐treated and untreated BCa cells showed an increase in mitochondrial localization of Bax. Moreover, the mitochondrial localization of pore factor Bcl‐2 decreased (Fig. 7C).

### Tannic acid induces intrinsic apoptosis

In the BCa cells, treatment with TA decreased the mitochondrial membrane potential (ΔΨm) in a concentration‐dependent manner (Fig. 7D, *P* < 0.001) in both p53‐mutated and wild‐type cells. The release of cytochrome c from mitochondria is also accompanied by a reduction in the ΔΨm (Fig. 7C). In addition, relatively significant levels of activated whole caspases were found in TA‐treated BCa cells (Fig. 7E). The inhibition of caspases with Z‐VAD‐fmk resulted in recovery from TA‐induced apoptosis, confirming that apoptosis was caspase‐dependent (Fig. 7F, *P* < 0.001). Collectively, these data confirm that the apoptosis induced by TA is intrinsic apoptosis.

## Discussion

Natural compounds are a possible source of molecules with anti‐proliferative effects in a broad range of cancers and may target specific signalling pathways to suppress tumours 36. Tannins and tannin‐containing foods have anti‐cancer activities against a broad spectrum of cancers, including BCa 2, 3. As previously documented, polyphenols and members of the tannin family have the ability to induce G1 arrest in various cancer cells 3. However, the mechanism underlying their action requires more attention.

Previous studies revealed that STAT1 serine phosphorylation requires Jak2 phosphorylation 43. Early time analysis showed that TA also induces Jak2 phosphorylation, and after 20 min. of treatment, it induces consistent inhibition of Jak2 phosphorylation. Inhibition of Jak2 using AG490 did not alter the level of STAT1 ser727 phosphorylation, but it partially recovered TA‐induced STAT1 tyr701 phosphorylation and G1 arrest. The p38 protein plays a critical role in G1 arrest 44, 45 and STAT1 serine phosphorylation 46. In this study, the induction of STAT1 ser727 phosphorylation by TA was p38‐dependent, as inhibition of p38 decreased ser727 phosphorylation. In addition, p38 activation is involved in STAT1 transcriptional functions, as DNA‐binding activity was inhibited upon p38 inhibition. We confirmed that TA‐induced STAT1 ser727 phosphorylation is Jak2‐independent and p38‐dependent. STAT1 ser727 and tyr701 phospho‐mutants revealed that ser727 and tyr701 residues are both important for the induction of G1 arrest upon TA treatment. The results of these experiments also show that the upstream regulatory tyrosine kinase Jak2 has no role in TA‐mediated G1 arrest, whereas the serine kinase p38 is critical in TA‐induced G1 arrest.

In nearly all mammalian cells, proliferation is controlled mainly in G1 phase and the cells automatically progress through the remaining phases 47. G1 arrest has been reported to be p53‐dependent and to rarely or not occur in p53‐mutated cells. Our present study using TA on p53 mutant (MDA‐MB 231, T47D) and p53 wild‐type (MCF‐7) BCa cells demonstrated prominent G1 arrest through a discrete p53‐independent mechanism that requires p21^Waf1/Cip1^ expression. Tannic acid treatment enhanced the expression of the principal downstream gene of p53, p21^Waf1/Cip1^, and induced G1 arrest. The role of STAT1 in cytokine‐induced cell arrest was reported previously; IFN‐γ induces cell cycle arrest through STAT1 48. We observed that TA directly controls p21^Waf1/Cip1^ expression through STAT1, which binds to the promoter of the p21^Waf1/Cip1^ gene. STAT1 also regulate the expression of p21^Waf1/Cip1^ and p27^Kip1^ and induce cell cycle arrest by blocking cyclins and CDKs. In this study, TA induced hyper‐activation of the p16^Ink4a^, p21^Waf1/Cip1^ and p27^Kip1^ genes. STAT1 silencing rescued cells from G1 arrest and resulted in a decrease in TA‐induced p21^Waf1/Cip1^ and p27^Kip1^ expression. Increase in STAT1 serine phosphorylation and decrease in STAT1 tyrosine phosphorylation can lead to the activation of p27^Kip1^ 49. Our experiments also showed an increase in p27^Kip1^ with increased STAT1 serine phosphorylation and decreased tyrosine phosphorylation. Studies with phosphorylation‐specific STAT1 mutants have shown that both serine and tyrosine are involved in TA‐induced p21^Waf1/Cip1^ and p27^Kip1^ expression. Taken together, the results indicate that distinct mechanisms and/or a dual combination of STAT1 ser/tyr phosphorylation are involved in the G1 arrest.

As we demonstrated here, TA binds to the ATP binding pocket of EGF‐R and inhibits its auto‐phosphorylation, resulting in the inhibition of Jak2 phosphorylation. Jak2 is the major upstream regulator of STAT3 phosphorylation 50 and the major mediator of the canonical STAT signalling pathway. Inhibition of Jak2 phosphorylation resulted in the inhibition of STAT3 phosphorylation. Generally, STAT3 is phosphorylated on ser727 and tyr705 residues 51, with tyr705 being responsible for the nuclear translocation and DNA‐binding activities of STAT3 2. Tannic acid inhibited the phosphorylation of both ser727 and tyr705. Analysis of nuclear extracts also confirmed the inhibition of STAT3 nuclear transport. One of the major functions of STAT3 is binding to its downstream target gene promoters and transcriptionally activating them. In this study, the DNA‐binding activity of STAT3 was inhibited by TA treatment, which was confirmed by a ChIP assay specific to the Bcl‐2 gene promoter.

Transcriptional analysis of STAT3 downstream targets, such as Bcl‐2 and Bcl‐X_L_, also confirmed the ability of TA to inhibit transcriptional activation by STAT3. The role of Bcl‐2 and Bcl‐X_L_ in TA‐induced apoptosis was confirmed by analysis of mitochondrial protein localization. In mitochondria, Bcl‐2 and Bcl‐X_L_ act as anti‐pore factors and inhibit the release of cytochrome c into the cytosol and apoptosis 52. Mitochondria isolated from TA‐challenged cells had reduced levels of both Bcl‐2 and Bcl‐X_L_, pointing to a loss of pore‐closing factors. Furthermore, Bax is highly localized on the mitochondria. Thus, these factors induced loss of ΔΨm and the release of cytochrome c into the cytosol. Cytochrome c is an activator of zymogenic caspases. Once the cytochrome c is released into the cytosol, it activates the procaspase to active caspase 53. We demonstrated the activation of poly caspases and that specific inhibition by Z‐VAD‐fmk rescued the cells from undergoing apoptosis, confirming the involvement of the mitochondrial pathway in TA‐mediated apoptosis.

Although the dosage of TA used in this study is relatively high, there are a lot of researches dealing with the *in vivo* administration of high dosage of TA. In an *in vivo* study conducted by Mori *et al*., a dosage of 30 mg/kg bw TA prepared in distilled water inhibited beta secretase and prevented the cognitive impairment and reduced Alzheimer like pathology 54. Similarly, in male albino rats, a dosage of 45.0 mg per 100 g bw (LD_50_ = 2260 mg/kg) daily administered for maintaining digestive tract microflora 55. In another study conducted by Seun *et al*., a dose of 40 mg/kg bw showed protective effect over cisplatin induced renal toxicity 56. A total of 25 mg/kg dosage of TA administered to the male Sprague‐Dawley rats also conferred protective effects from the acetaminophen induced acute hepatic toxicity 57.

The applicability of TA in the treatment of cancer also includes its ability to synergize the drug activities. In this study, TA synergized the action of TAM in ER‐positive BCa cells. Moreover TA synergized the activity of GEF in TNBC cells. As the TNBC cells lack the nuclear and other hormonal receptors, EGFR is a primary target for drug action, though, GEF needed to use in higher concentration to achieve desired proliferation inhibition in MDA‐MB 231 cells. In this study, with the combination of TA, GEF concentration reduced to a better extend with no alteration in the potency. In human cholangio carcinoma, TA modulated the drug efflux pathways and synergized the cytotoxicity of various chemotherapeutic drugs 11. Hence, the usage of TA as an adjunct to chemotherapy is warranted.

In conclusion, this study shows that TA has the ability to regulate the canonical EGF‐R/Jak2/STAT pathway and non‐canonical p38/STAT1 pathway. For the induction of G1 arrest, TA used the non‐canonical p38/STAT1/p21^Waf1/Cip1^ signalling axis with STAT1 ser727 as the critical factor. For the induction of apoptosis, TA used the canonical EGF‐R/Jak2/STAT3/BCl‐2 signalling axis. The results point the role of TA in gene‐specific regulation, and induction of G1 arrest and apoptosis. Moreover, TA also showed the ability to synergize drug action. Therefore, we recommend the nutraceutical TA or tannin‐containing foods as critical tumour therapeutic and preventive agents.

## Conflict of interest

The authors declare that there is no conflict of interest exists.

## Author contribution

PD and YMY conceived and designed the experiments. PD, HS, YHJ, DYK, NSP and HJB performed the experiments. PD, HS, YMY, YHJ, TSH, CHL, KHC, KDP and HKL analysed the data and evaluated the manuscript. PD and YMY wrote the manuscript.

## Supporting information


**Figure S1** TA binds to the ATP binding domain of tyrosine kinases. The molecular docking study was done to different tyrosine kinases. The ATP binding domain of the receptors was docked with the ligand, TA using the autodock vina platform. (**A**) TA binding with Insulin receptor (IR, PDB ID for the molecule, 1IRK). (**B**) TA binding to the IGF‐1R (IDB ID: 1JQH). (**C**) TA binding to the Jak‐2 (PDB ID: 2B7A). (**D**) TA binding to the Jak‐1 (PDB ID: 4K6Z).
