# Supplementary figures and images for: Tannic acid inhibits EGFR/STAT1/3 and enhances p38/STAT1 signalling axis in breast cancer cells
Source: J Cell Mol Med. 2016 Nov 15;21(4):720–34. doi: 10.1111/jcmm.13015 (PMC5345631; doi:10.1111/jcmm.13015)

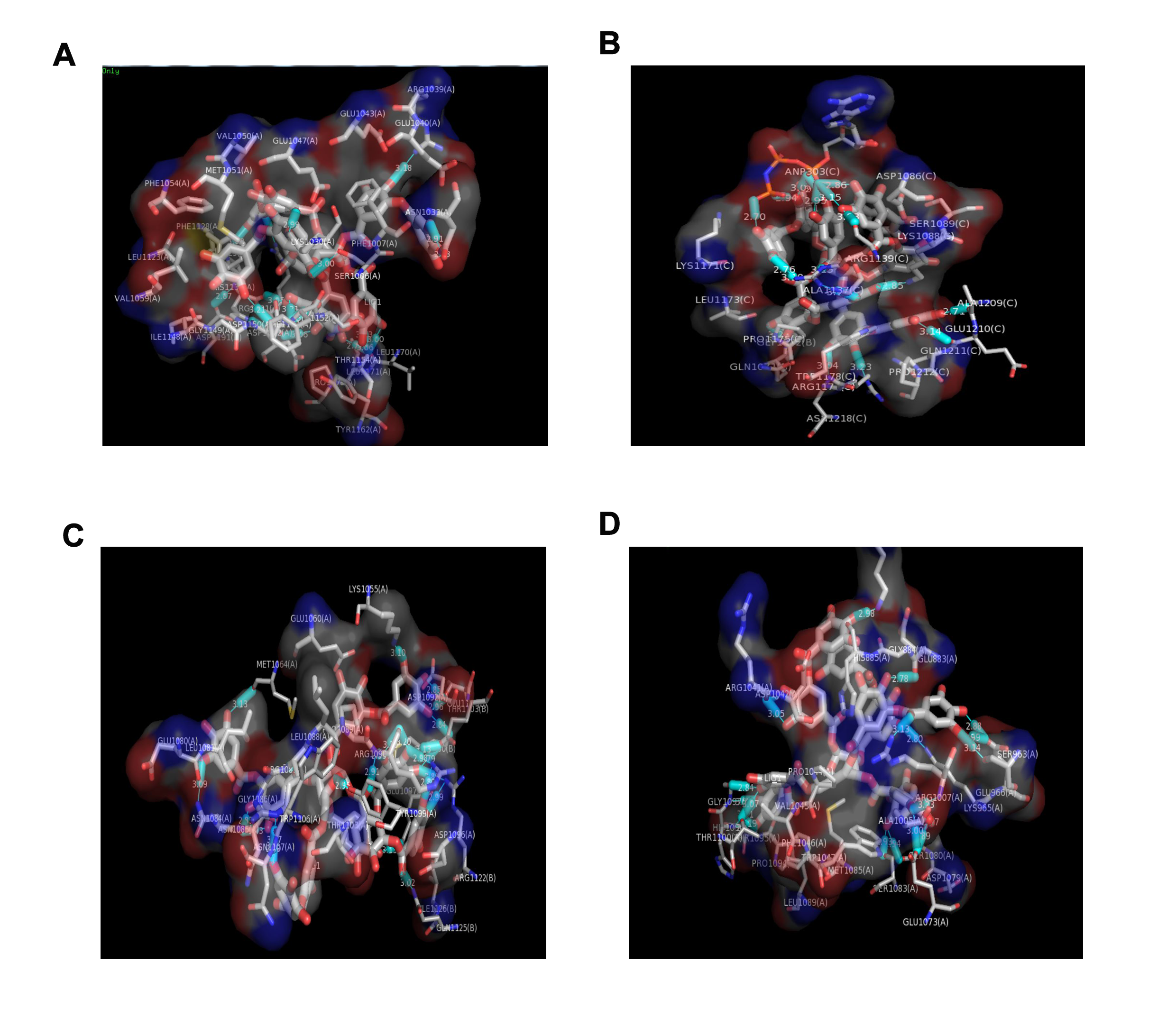

Supplement: Supplementary file 1 — Figure S1 TA binds to the ATP binding domain of tyrosine kinases. The molecular docking study was done to different tyrosine kinases. The ATP binding domain of the receptors was docked with the ligand, TA using the autodock vina platform. (A) TA binding with Insulin receptor (IR, PDB ID for the molecule, 1IRK). (B) TA binding to the IGF‐1R (IDB ID: 1JQH). (C) TA binding to the Jak‐2 (PDB ID: 2B7A). (D) TA binding to the Jak‐1 (PDB ID: 4K6Z). [file JCMM-21-720-s001.tif]
